# Keratinocytes Propagated in Serum-Free, Feeder-Free Culture Conditions Fail to Form Stratified Epidermis in a Reconstituted Skin Model

**DOI:** 10.1371/journal.pone.0052494

**Published:** 2013-01-11

**Authors:** Rebecca Lamb, Carrie A. Ambler

**Affiliations:** 1 School of Biological and Biomedical Sciences, Durham University, South Road, Durham, United Kingdom; 2 Biophysical Sciences Institute, Durham University, South Road, Durham, United Kingdom; University of Newcastle upon Tyne, United Kingdom

## Abstract

Primary human epidermal stem cells isolated from skin tissues and subsequently expanded in tissue culture are used for human therapeutic use to reconstitute skin on patients and to generate artificial skin in culture for academic and commercial research. Classically, epidermal cells, known as keratinocytes, required fibroblast feeder support and serum-containing media for serial propagation. In alignment with global efforts to remove potential animal contaminants, many serum-free, feeder-free culture methods have been developed that support derivation and growth of these cells in 2-dimensional culture. Here we show that keratinocytes grown continually in serum-free and feeder-free conditions were unable to form into a stratified, mature epidermis in a skin equivalent model. This is not due to loss of cell potential as keratinocytes propagated in serum-free, feeder-free conditions retain their ability to form stratified epidermis when re-introduced to classic serum-containing media. Extracellular calcium supplementation failed to improve epidermis development. In contrast, the addition of serum to commercial, growth media developed for serum-free expansion of keratinocytes facilitated 3-dimensional stratification in our skin equivalent model. Moreover, the addition of heat-inactivated serum improved the epidermis structure and thickness, suggesting that serum contains factors that both aid and inhibit stratification.

## Introduction

The human skin is a stratified, multi-layer epithelium that has an extensive capacity for self-renewal and tissue repair [1], [2]. When surface cells are lost due to routine sloughing or damage, naïve progenitor cells located in the basal layer of the epidermis leave the basal surface and start a stepwise and orchestrated programme of differentiation to replenish lost, terminally differentiated layers. Proliferative, undifferentiated keratinocytes can be isolated from juvenile and adult human skin and stimulated to differentiate *in vitro* by adding exogenous factors including calcium, serum or the phorbal ester, TPA, by inhibiting keratinocyte-extracellular matrix contact through forced cell suspension or by seeding primary epidermal keratinocytes onto dermal equivalent substrates grown at the air-liquid interface [3]–[7]. Only the latter supports epidermal stratification. Growing primary epidermal keratinocytes at the air-liquid interface on dermal equivalent substrates, like de-epidermalised, de-vitalised human skin (DEDs) mimics the natural skin environment. DEDs retain extracellular matrix proteins and tissue architecture (undulating rete-ridge patterning) of vital dermis. Keratinocytes grown under these 3-dimentional promoting conditions differentiate into a fully stratified epidermis with basal, spinous, granular and cornified cell layers [7]. This DED skin equivalent model is used widely in both academic and commercial research [8], [9].

Routine methods for the *in vitro* culture and clonal expansion of primary keratinocytes were first developed in the 1970's by James Rheinwald and Howard Green. These methods rely on co-culture of human keratinocytes with irradiated mouse J2-3T3 fibroblast feeders in serum-containing basal medium supplemented with growth factors (i.e. insulin, hydrocortisone, colera toxin and epidermal growth factor) [10]. Modified Rheinwald and Green culture conditions are still extensively used today and are compatible with autologous and allogenic transplantation [11], [12]. Nevertheless, in line with the extensive efforts to ensure that cells grown for human therapeutic use are free of potential harmful agents like bovine spongio encelopathy and some mouse viruses, new defined, serum-free and feeder-free growth methods have been developed in the last decade [13], [14]. Successful efforts from Sheila MacNeil's laboratory and others include the development of methods for derivation and growth of keratinocytes in serum-free media and the use of human, rather than murine fibroblast feeders to support expansion of undifferentiated human keratinocyte under serum-free conditions and skin reconstitution in an *in vitro* wound bed model [15]–[17]. Numerous commercial companies have developed serum-free, feeder-free media that support keratinocyte propagation and expansion in 2-dimensional tissue culture, but how these growth conditions affect the ability of keratinocytes to stratify and differentiate in 3-dimensional skin epidermis has not been fully investigated.

Here we demonstrate that two serum-free, feeder-free growth media sufficient for propagation of primary epidermal keratinocytes do not support epidermis development in an *in vitro* skin equivalent model. This insufficiency in the media is not caused by reduced Ca^++^ levels as addition of exogenous Ca^++^ failed to rescue epidermis development. However, a stratified epidermis will form using serum-free media supplemented with 10% fetal-bovine serum (FBS) at both high and low Ca^++^ concentration. Stratified epidermis forms with the addition of heat-inactivated but not boiled serum, indicating that serum factors required for *in vitro* epidermis development are likely to be biologically-active.

## Methods

### Ethics Statement

In this study, de-epidermalised, de-vitalised dermal substrates were generated from whole human skin. Human skin samples were collected under a UK National Research Ethics Service study protocol (05/MRE01/72) approved by the local Durham University ethics committee and the Northern and Yorkshire Research Ethics Committee. Written, informed patient consent was obtained for all samples.

### Cell Culture

Third passage Neonatal Human Epidermal Keratinocytes (NHEKs Lonza) were initially cultured in serum-free conditions in Lonza's KGM-Gold media (basal medium supplemented with bovine pituitary extract, human epidermal growth factor, bovine insulin, hydrocortisone, Gentamicin, Amphotericin-B, Epinephrine and Transferrin) in collagen coated (1∶100; Type 1 rat tail collagen (Sigma) : PBS) T25 flasks. The cells were cultured in a 37°C incubator with 5% CO_2_, with the media being changed every 2 days until approximately 80% confluent. The NHEK's were then lifted with 3∶1 PBS/trypsin-EDTA (0.25%), which was neutralised by adding KGM-Gold media with 10% FBS before cells were centrifuged at 200× g for 5 minutes and counted with a hemocytometer. Cells were seeded into either a collagen coated T25 flask containing KGM-Gold or a collagen coated T25 flask using the method of Rheinwald and Green [10] at a density of 100000 cells per flask. The Rheinwald and Green method involves culturing the NHEKs on a mitotically inactivated feeder layer (JT-3T3 cells) in complete FAD media (made from 3 parts Dulbecco's modified Eagle's medium (DMEM) to 1 part Ham's F12 medium (F12) supplemented with 1.8×10^−4^ M adenine, 100 IU/ml penicillin, 100 µg/ml streptomycin, 10% fetal bovine serum, 0.5 ug/ml hydrocortisone, 8.47 ng/ml cholera enterotoxin, epidermal 10 ng/ml growth factor and 5 µg/ml insulin.

Four different NHEK primary cell lines (Lines 22, 51, 76 and 06) were used to perform all experiments. Experimental repeats were performed using 3 or more different lines.

### Skin Reconstructions on De-vitalised dermis

Once confluent, fourth passage NHEKs were lifted, counted and used to create an artificial epidermis on a section of DED. DEDs were prepared using the following method; skin was sterilised using 10% Videne (Ecolab) then heat-treated to remove the epidermis and cut into approximately 1 cm^2^ pieces. The pieces of skin were freeze-thawed 10 times to ensure no viable host cells remained. When required, a piece of DED was thawed, trimmed and placed on a well insert which sat in a 6-well plate (Greiner). Onto one piece of DED, 300000 NHEKs were seeded and 1 ml of media was placed in the well under the insert.

The following cell culture media were used: KGM-Gold (Lonza; 0.1 mM Ca^++^), complete FAD (1.4 mM Ca^++^), low-Ca^++^ FAD (0.05 mM Ca^++^) or EpiLife (Gibco; 0.06 mM Ca^++^). Some media were supplemented with fetal bovine serum (A15-108 PAA Laboratories) and/or calcium (as calcium chloride) (see results for details). When required, serum was heat inactivated in a 56°C water bath for 30 mins or boiled at 96°C for 10 mins.

To analyse skin development, DED cultures were incubated for 14 days before the DED was removed and bisected. One-half was frozen in OCT (Thermo Scientific Raymond Lamb) and subsequently cryosectioned. The other half was fixed in 4% paraformaldehyde overnight at 4°C before being embedded in paraffin and sectioned on a microtome (8 µm-thick sections).

### Tissue Labelling

Histology of tissue sections was visualized using hematoxylin and eosin (H&E) staining as previously described [18]. For immunofluorescent detection, paraffin-embedded tissue sections were de-paraffinised in xylene, then rehydrated in a graded alcohol/water series. Antigen-recovery was performed by microwaving sections in 10 mM tri-sodium citrate (pH 6.0) for 10 minutes. The samples were blocked for 1 hour at room temperature (RT) in a blocking serum (10% goat serum/0.25% Fish Skin Gelatin/0.2% BSA/PBS) and then incubated with a suitable dilution of the primary antibody for 1–2 hrs at RT. Next, slides were rinsed, 3 times with PBS for 5 minutes then incubated with a species-specific Alexa-conjugated secondary antibody (1∶1000, Life Technologies) with DAPI (1∶10000) for 40 mins at RT, and then mounted with Mowiol (Mowiol 4.88, Calbiochem catalog number 475904) [19].

### Calculating the Depth of the Epidermis

Images of H&E stained DED sections and of a S8 stage micrometer graticule were photographed using a Leica MZ125 microscope with a JVC camcorder all set at the same zoom. To determine average epidermal thickness, images were analysed using ImageJ and the scale calibrated to the graticule image. The total epidermis on each section was outlined by free hand drawing around the epidermal layers, and divided by the total length of the DEDs to produce an average depth of the epidermis in microns. This process was repeated for all the DEDs using the different NHEK cell lines to obtain the mean epidermis depth for all the different types of media. The standard error of the mean (s.e.m) and statistical significance (Student's t-test) was determined using GraphPad statistical software.

### *Culture cells for histology*

Collagen coated glass cover slips were placed into each well of a 24-well plate. Some cover slips were seeded with 100000 mitotically inactivated JT-3T3 feeder cells, before adding 15000 NHEKs. Cells were grown in complete FAD or serum-free KGM-Gold media for 48 hrs at 37°C then fixed in 4% PFA for 5 minutes and washed with PBS. Staining was performed on the cells by blocking the cover slips in 0.2% Fish skin gelatin/PBS for 30 mins at RT and then permeabilising with 0.2% Tween-20/PBS for 5 minutes. To stain actin and the nucleus, coverslips were incubated with phalloidin-rhodamine and DAPI for 15 mins at RT. The slides were mounted with Mowiol and imaged immediately.

### Live cell imaging

Live cultures were imaged every 20 minutes for 6–24 hours using a Zeiss Axiovert 200M microscope at 37°C and 5% C0_2_. Images were compiled into a video sequence using ImageJ analysis software. Cell (n = 10) motility was tracked using a MtrackJ plugin on ImageJ and the data was represented on a scatter graph with all the start positions normalized to zero.

### Antibodies and Stains

Primary antibodies and cell stains used were Anti-Involucrin antibody 1∶500 (Abcam, ab27495), Keratin 14 Polyclonal Antibody 1∶1000 (Covance, PRB-155P), Keratin 10 Polyclonal Antibody 1∶1000 (Covance, PRB-159P), Molecular Probes rhodamine phalloidin 1∶200 (Life Technologies, R415) and DAPI 1∶10000 (Life Technologies, D3571). Stained slides and coverslips were visualised using a Zeiss Axioskop 40 microscope, Zeiss AX10 Imager M1 or Leica SP5 confocal microscope.

## Results and Discussion

We first examined what happens when primary epidermal cells isolated and maintained in serum-free conditions are subsequently introduced to traditional feeder-supported cell culture. Four lines of primary, neonatal epidermal keratinocytes (Lines 06, 22, 51 and 76) were purchased from a commercial source. Cells were derived and maintained continuously in serum-free, feeder-free conditions and P3 or P4 cells were used for this and all subsequent experiments. To confirm efficacy of the results experiments were repeated using a minimum of 3 independent cell lines.

**Figure 1:**
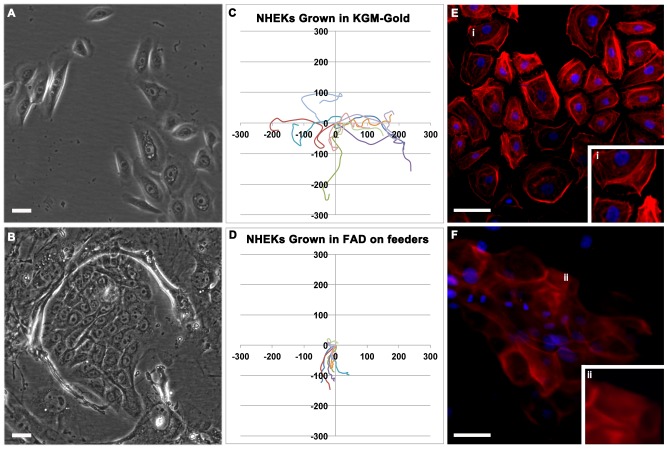
Keratinocyte behaviour in monolayer cultures. (**A–D**) Live cell images of p3 NHEKs grown in standard tissue culture flasks. Cells were grown in either KGM-Gold, serum-free media (A,C) or in complete FAD with feeder-support (B,D). (**C,D**) Trace of individual cells over a period of 24 hours. Axis scale in µM. (**E,F**) Equal numbers of p4 NHEKs were seeded onto cover-slips in a 24 well plate and grown in either KGM-Gold, serum-free media (E) or in complete FAD with feeder-support (F). Once confluent, the cells were fixed and then stained with phalloidin (red) and DAPI (blue). Scale bars: 50 microns.

Trypsinised cells were plated at equal density in KGM-Gold, serum-free media (0.1 mM Ca^++^) or in growth-factor supplemented, complete FAD (1.4 mM Ca^++^ and 10% fetal bovine serum) with feeder-support (Figure 1). 48 hours after plating, cell motility was analysed by live-cell imaging (Figure 1A, B, C, D and Movies S1 and S2). In KGM-Gold, keratinocytes were distributed throughout the culture as single cells or localised in small, loosely-connected groups. Calcium is needed to form desmosomes and cell junctions [20]–[22] and thus, cells grown in low-Ca^++^ KGM-Gold media were highly motile with ruffling lamellipodia and formed temporary cell attachments (Figure 1C). Cells grown in high-Ca^++^ compete FAD formed tight clusters that moved in the cell dish as a connected group (Figure 1D). Cells remained uniform and undifferentiated within colonies. To visualise the actin filamentous network, cells were fixed and stained with phalloidin. Consistent with motile nature of cells grown in low-Ca^++^ KGM-Gold, polymerised actin was detected in lamellipodia at the leading edge of the cell (Figure 1C), whereas actin staining was uniform in cells grown on feeders in complete FAD (Figure 1D). These results show that keratinocytes grown in low-Ca^++^, serum-free conditions revert to classic, colony-forming growth habit when placed in feeder-dependent, high-Ca^++^ growth conditions.

**Figure 2:**
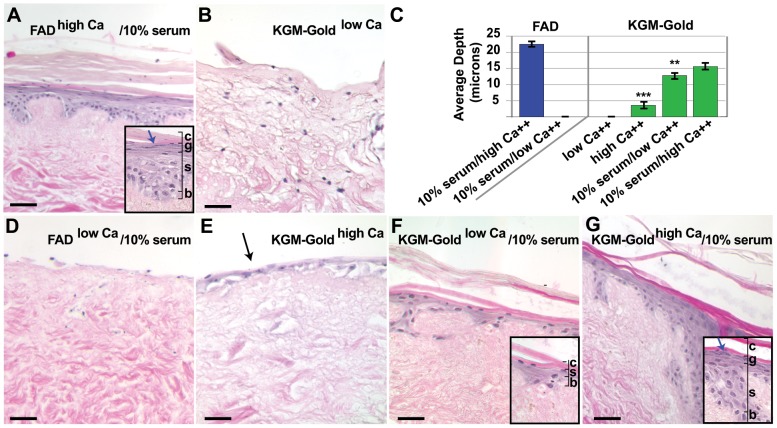
Keratinocyte media sufficient to support feeder-free, serum-free epidermal growth fails to support stratification in skin equivalents. (**A, B, D–F**) Hematoxylin and eosin-stained sections of p4 NHEKs grown on DEDs in the following culture media: (A) complete FAD (high Ca^++^/10%FCS), (B) KGM-Gold (low Ca^++^), (D) low-Ca^++^ FAD with 10% serum (low Ca^++^/10%FCS), (E) KGM-gold plus added Ca^++^ (high Ca^++^), (F) KGM-Gold supplemented with 10% fetal bovine serum (low Ca^++^/10%FCS) and (G) KGM supplemented with 10% fetal bovine serum and added Ca^++^ (high Ca^++^/10%FCS). Insets show higher magnification images. Layers of the stratified epidermis indicated: B, basal; S, spinous; G, granular; C, cornified. (**C**) Graph indicates the average depth of the epithelium layer in the different types of media. Statistical significance calculated relative to control, complete FAD (high Ca^++^/10%FCS) cultured samples. Black arrow in B marks keratinocytes on DED surface. Blue arrows in A and G marks granular cells. ** p value<0.005; *** p value<0.0005 (Student's t-test); scale bars: 50 microns.

Next, we tested if keratinocytes grown in serum-free media would stratify and differentiate into a multi-layered skin equivalent when grown on de-vitalised, de-epidermalised dermis (DED) at the air-liquid interface. Keratinocytes isolated and maintained in serum-free KGM-Gold media were added to the DED surface at the air-liquid interface and subsequently grown in KGM-Gold or complete FAD for 14 days (Figure 2A,B). Hematoxylin and eosin-stained tissue sections revealed a thick, multi-layered epidermis comprised of basal, suprabasal (spinous and granular) and cornified layers on DED tissues grown in FAD, but not KGM-Gold media (Figure 2A, B). We quantified average epidermal thickness of basal and suprabasal layers not including the surface cornified material from experimental replicates (n≥4) using three or four independent cell lines (Figure 2C). Average epidermal thickness on DED cultures grown in complete FAD was 22.4 microns±0.74 (s.e.m.). Comparibly, no epidermis formed on the surface of DEDs grown in KGM-Gold and only a small number of nucleated cells were detected (Figure 2B). Importantly, these results show that early passage primary keratinocytes isolated and maintained in serum-free conditions can stratify under the right conditions, therefore the inability of keratinocytes to stratify and differentiate in serum-free media is due to a media insufficiency rather than a loss of overall cell potential.

We wanted to further investigate why complete FAD media, but not KGM-Gold was able to support formation of a stratified epithelia on DED tissues. Both medias contain similar growth-factor supplements so we focused on two obvious differences between these media, Ca^++^ concentration and addition of 10% serum to the FAD media. Extracellular calcium is required for epithelial stratification [23] and in our DED-model of 3-D reconstituted skin, low-Ca^++^ FAD medium (0.05 mM) containing 10% serum is not sufficient to support the differentiation and stratification of keratinocytes in 3-D culture (Figure 2D). As complete FAD has relatively high levels of Ca^++^ (1.4 mM) compared to KGM-Gold (0.1 mM), we tested if raising Ca^++^ concentration of KGM-Gold media to 1.4 mM was sufficient to support epidermis development (Figure 2E). H and E stained tissues revealed a thin epidermal layer at the surface with 1–2 layers of nucleated cells, but no surface cornified material was detected. Epidermis was significantly thinner (3.6 microns±0.95 s.e.m.; p<0.0001) compared to controls grown in complete FAD.

Next, we tested the role of serum. We grew keratinocyte-seeded DED cultures in low-Ca^++^ KGM-Gold medium supplemented with 10% serum. Layered keratinocytes were evident with a thin cornified layer over the epidermal surface. However, epidermal thickness was approximately half (12.8 microns±1.10 s.e.m.; p = 0.0017) that of controls grown in complete FAD (Figure 2F). Also, in histological-stained sections, the suprabasal granular layer could not be detected. The characteristic feature of the granular layer is the distinctive presence of keratohyalin granules readily detectable by histological staining (Figure 2A). In DEDs cultured in serum-supplemented KGM-Gold, the presence of evident surface cornified material despite the lack of an evident granular layer suggests that keratinocytes are undergoing abnormal differentiation.

However, a fully-stratified epidermis including the suprabasal granular layer with detectable keratohyalin granules formed on DEDs cultured in KGM-Gold media supplemented with both exogenous Ca^++^ and 10% serum (Figure 2G). Moreover, addition of both supplements significantly increased overall epidermal thickness (15.6 microns±1.15 s.e.m.; p = 0.0209) compared to KGM-Gold cultured controls.

**Figure 3:**
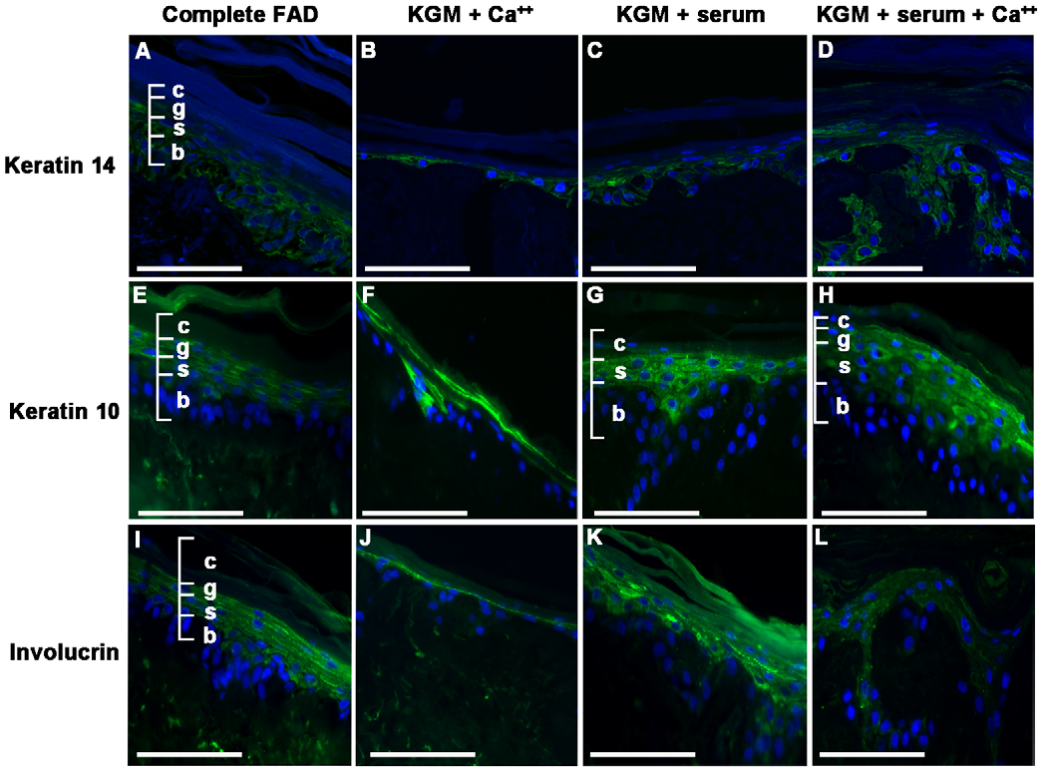
Expression of keratinocyte differentiation markers. (**A–L**) Eight micron sections of NHEKs grown on DEDs stained with antibodies to Keratin 14 (A–D, green), Keratin 10 (E–H, green) and Involucrin (I–L, green). All sections were counterstained with DAPI (blue). DEDs were grown in complete FAD (A,E,I), KGM-Gold plus added Ca^++^ (high Ca^++^; B,F,J), KGM-Gold supplemented with 10% fetal bovine serum (low Ca^++^/10%FCS; C,G,K) or KGM supplemented with 10% fetal bovine serum and added Ca^++^ (high Ca^++^/10%FCS; D,H,L). Layers of the stratified epidermis indicated: B, basal; S, spinous; G, granular; C, cornified. Scale bars: 50 microns.

As naïve keratinocytes differentiate they down-regulate a characteristic set of basal proteins and up-regulate suprabasal markers [2], [24]. To investigate keratinocyte differentiation in various growth media we stained sections of DEDs grown in complete FAD or in KGM-Gold with added Ca^++^, 10% fetal bovine serum or both with antibodies to keratin 14 and the suprabasal markers, keratin 10 and involucrin (Figure 3A, B, C, D, E, F, G, H, I, J, K, L). Keratin 14, keratin 10 and involucrin were detectable in all samples. DEDs cultured in KGM-Gold supplemented with only Ca^++^ had fewer suprabasal layers and no cornified layer, thus there was a corresponding reduction in keratin 10 and involucrin expression (Figure 3B, F, J). We found a characteristic hierarchy of expression with keratin 14 detectable in both basal and suprabasal layers and keratin 10 and involucrin detectable in suprabasal cells irrespective of culture media and Ca^++^ level. Keratin 10 and involucrin proteins are some of the first proteins to be expressed when cells commit to differentiate. In fact, involucrin can be detected on some basal cells within intact skin tissues and some non-stratified primary keratinocytes [24]–[26]. *In vitro*, Ca^++^ can induce differentiation, however it has been shown that other environmental factors, including cell confluency, stimulate keratinocytes to differentiation in both high and low calcium media [27]. Similarly, our results suggest that additional, Ca^++^-independent factors regulate involucrin and keratin 10 expression in differentiating keratinocytes grown on DEDs at the air-liquid interface.

**Figure 4:**
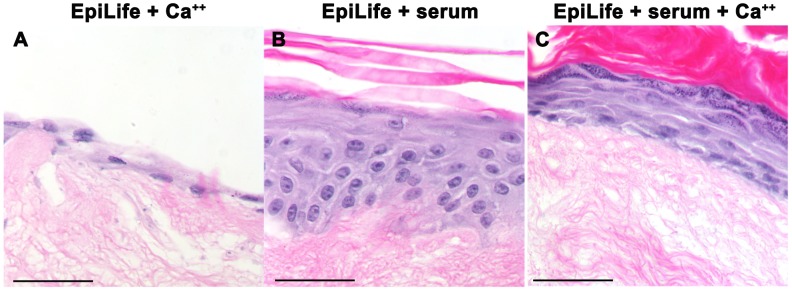
EpiLife media supplemented with calcium and 10% serum supports keratinocyte stratification. (**A–C**) Hematoxylin and eosin-stained sections of p4 NHEKs grown on DEDs in the following culture media: (A) EpiLife plus added Ca^++^ (high Ca^++^), (B) EpiLife supplemented with 10% fetal bovine serum (low Ca^++^/10%FCS) or (C) EpiLife supplemented with 10% fetal bovine serum and added Ca^++^ (high Ca^++^/10%FCS). Scale bars: 50 microns.

We tested a second serum-free, low Ca^++^ growth media, EpiLife, for its ability to support epidermis development in our *in vitro* models. Like, KGM-Gold, EpiLife media supports the derivation and expansion of primary epidermal cells *in vitro* [28]. However, we found that EpiLife also failed to support stratification and differentiation of keratinocytes on DEDs in the absence of serum (Figure 4A). Interestingly, unlike DEDs grown in serum-supplemented KGM-Gold, keratohyalin-containing granular cells were detectable by histological staining in the epidermis formed on DEDs cultured in serum-supplemented EpiLife medium (Figure 4B). However the epidermis looked abnormal; the epidermis was thin and lacked small, tightly packed basal cells. Only adding both 10% serum and exogenous Ca^++^ (final 1.4 mM) to EpiLife media resulted in a thick, well structured epidermis with basal, undifferentiated cells and spinous and granular layers detected by histological staining (Figure 4C) compared to media with either 10% serum or exogenous Ca^++^ added. Taken together these results show that serum-free keratinocyte growth media supplemented with 10% serum and sufficient free Ca^++^ ions facilitates epidermal stratification and terminal differentiation in 3-dimensional *in vitro* skin models.

**Figure 5:**
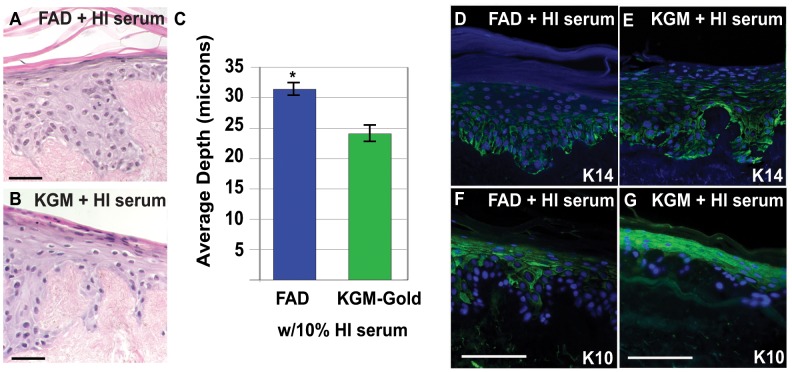
Heat-treating serum increases epidermal thickness and organisation. Eight micron sections of p4 NHEKs grown on DEDs in complete FAD with 10% serum heated to 56°C (A, D, F; FAD+HI serum) or KGM supplemented with 10% serum heated to 56°C and added Ca^++^ (B, E, G; KGM+HI serum). (**A, B, D–H**) Sections were stained with hematoxylin and eosin or with antibodies to Keratin 14 (D, E, green) or Keratin 10 (F, G, green). Sections were counterstained with DAPI (blue). (**C**) Graph indicates the average depth of the epithelium layer in the two types of media. Statistical significance calculated relative to control, complete FAD with 10% serum (high Ca^++^/10%FCS) cultured samples (Figure 2). ** p value<0.005; *** p value<0.0005 (Student's t-test); scale bars: 50 microns.

**Figure 6:**
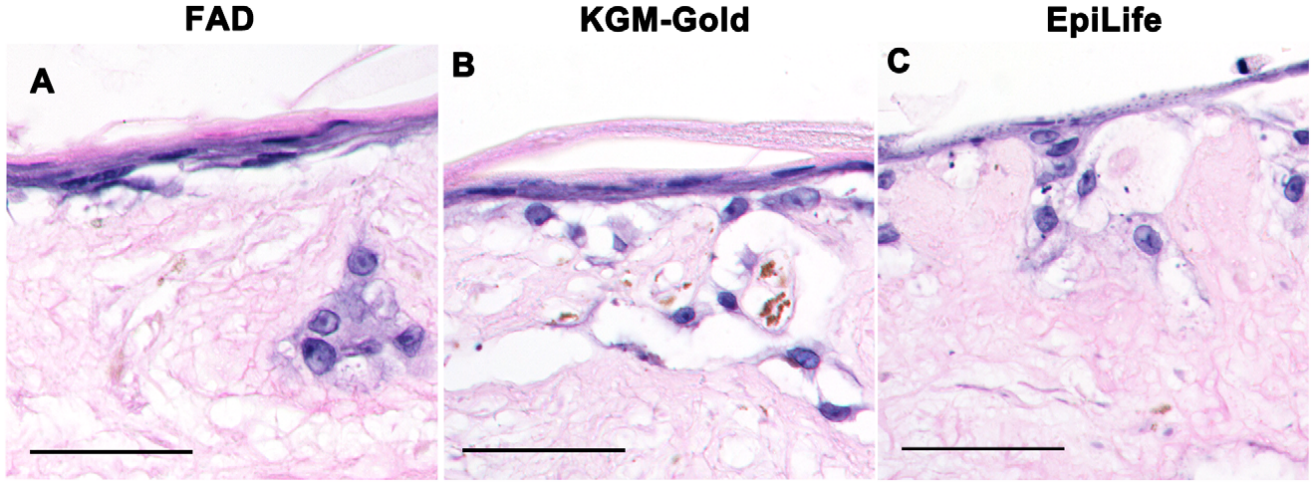
Heat-denaturation removes key serum factors needed to support epidermal stratification. (**A–C**) Eight micron sections of p4 NHEKs grown on DEDs in complete FAD (A), KGM-Gold (B) or EpiLife (C) supplemented with 10% serum boiled at 95°C and 1.4 mM Ca^++^. Scale bars: 50 microns.

To determine if complement proteins within the serum are needed for stratification, we inactivated complement proteins and other heat-sensitive factors in serum by heating fetal bovine serum to 56°C for 30 minutes before adding to the basal FAD or KGM-Gold (1.4 mM Ca^++^) growth medium [29]. Keratinocytes grown on DEDs in FAD or KGM-Gold supplemented with heat-inactivated serum differentiated into a thick, multi-layered epithelium (Figure 5A, B) with appropriate basal and suprabasal markers (Figure 5D, E, F, G and data not shown). Moreover, the epidermis was significantly thicker when DEDs were cultured in media containing heat-inactivated serum compared to the respective media with native serum (FAD: 29.9 microns±1.90 s.e.m.; p = 0.0068; KGM-Gold: 22.4 microns±1.47 s.e.m.; p = 0.0172). (Figure 2C and 5C). Having shown that complement factors sensitive to heat inactivation are not required for stratification we wanted to confirm if other biologically active serum factors are required by denaturing other serum proteins; serum heated to 96°C for 30 minutes then cooled was added to FAD, KGM-Gold or EpiLife media. Keratinoctyes grown on DEDs in these media failed to form a fully stratified epidermis (Figure 6A, B, C). These results show that mild heating of serum to inactivate complement and other heat sensitive factors did not inhibit the ability of serum to promote stratification, rather increased epidermal thickness; mild heat-treatment may inactivate a factor that inhibits differentiation *in vitro*. However, serum-induced stratification does require biologically active proteins as denaturing all serum proteins inhibits differentiation *in vitro*.

In summary, our data shows that isolated keratinocytes grown continually in serum-free and feeder-free conditions were unable to form into a stratified, mature epidermis *in vitro* under these conditions. We tested two, commercially-available, serum-free keratinocyte growth media (KGM-Gold and EpiLife) and both failed to stimulate epidermis development in a 3-dimensional skin equivalent model (DEDs) commonly used for drug and cosmetic research. Conversely, we found keratinocytes cannot be maintained on DEDs grown in serum-free media for 14 days. This is due in part to low levels of Ca^++^ in the basal media; epidermal keratinocytes form a detectable layer on the DED surface when cultured in serum-free media with exogenous Ca^++^ to a final 1.4 mM concentration. These observations are consistent with the known role of Ca^++^ as a factor important for inter-cellular junctions and hemi-desmosomes, which attach basal keratinocytes to the underlying extracellular matrix [20], [30]. However, we found the addition of exogenous Ca^++^ to serum-free media was unable to support the development of a fully-stratified epidermis. In our experiments, only the addition of serum to commercial serum-free growth media facilitated stratification in our skin equivalent model. Furthermore, we found that both serum and exogenous Ca^++^ supplements were required for formation of a detectable granular layer, one of the upper layers of normal stratified skin. The ultimate outcome of terminal differentiation is the strateum corneum, the skin's water impervious barrier. To be an effective barrier, lipids and proteins must be tightly organised and effectively cross-linked through progressive differentiation of keratinocytes first forming spinous, then granular cells before terminally differentiating into mature corneocytes [31].

It is well established that supporting fibroblast feeders provide nutritional support for keratinocyte growth [32]. Moreover, evidence suggests that fibroblasts also provide key factors needed for differentiation; keratinocytes form fully stratified skin in serum-free media when seeded onto fibroblast-coated DEDs or on collagen gels embedded with supporting fibroblasts - an alternative skin equivalent model [13], [33], [34]. However, our work shows that fibroblasts feeder support is not absolutely required; keratinocytes that have never been co-cultured with fibroblasts or exposed to fibroblast-conditioned media will form stratified skin in serum-supplemented media.

Serum, as a natural biological product, is a complex mixture of over one thousand known nutrients and proteins that is incompletely defined and varies depending on the donor [35]. We found a significant increase in epidermal thickness when serum heated to inactivate heat sensitive factors, like complement proteins, was used. This suggests that serum, although clearly important for promoting differentiation and stratification, may also contain inhibitory factors that are inactivated through heat-treatment. Moreover, Jean et al., 2011 showed that keratinocytes differentiated on a fibroblast feeder-containing, skin-equivalent model, the lipid organisation of the stratum corneum was better when cultures were serum-starved. Taken together these results suggest that key serum factors both aid and inhibit stratification. Further this leaves open the possibility of generating new media supplements specifically targeted for growth and development of 3-dimensional reconstructed tissues.

## Supporting Information

Live epidermal keratinocytes were imaged every 20 minutes for 6 hours using a Zeiss Axiovert 200M microscope at 37°C and 5% C0_2_. Images were compiled into a video sequence using ImageJ analysis software.

Movie S1 **Keratinocytes grown in KGM-Gold growth media.**
(MOV)

Movie S2 **Keratinocytes grown with fibroblast-feeder support in complete FAD media.**
(MOV)
